# Lessons from the Thai Health Promotion Foundation

**DOI:** 10.2471/BLT.18.220277

**Published:** 2018-12-19

**Authors:** Suladda Pongutta, Rapeepong Suphanchaimat, Walaiporn Patcharanarumol, Viroj Tangcharoensathien

**Affiliations:** a International Health Policy Program, Ministry of Public Health, Muang, Nonthaburi 11000, Thailand.

## Abstract

To facilitate the policy response to noncommunicable diseases in Thailand, parliament adopted the Health Promotion Foundation Act in 2001. This Act led to the establishment of an autonomous government body, the Thai Health Promotion Foundation, called ThaiHealth. The foundation receives its revenue from a 2% surcharge of excise taxes on tobacco and alcohol. The fund supports evidence generation, campaigns and social mobilization to address noncommunicable disease risk factors, such as tobacco-use, harmful use of alcohol and sedentary behaviour. On average, its annual revenue is 120 million United States dollars (US$). Some notable ThaiHealth-supported public campaigns are for schools free of sweetened carbonated beverages; alcohol abstinence during three-month Buddhist lent; and nationwide physical activity. The percentage of people using tobacco decreased from 22.5% in 2001 to 18.2% in 2014. The annual per capita alcohol consumption decreased from 8.1 litres pure alcohol in 2005 to 6.9 litres in 2014. The percentage of the adult population doing at least 150 minutes of moderate-intensity or 75 minutes high-intensity aerobic exercise per week, increased from 66.3% in 2012 to 72.9% in 2017. A dedicated funding mechanism, a transparent and accountable organization, and the engagement of civil society organizations and other government agencies have enabled ThaiHealth to run these campaigns.

## Résumé

Afin de soutenir l'action politique concernant les maladies non transmissibles en Thaïlande, le Parlement a adopté une loi sur la Fondation pour la promotion de la santé en 2001. Cette loi a conduit à l'établissement d'un organisme gouvernemental autonome, la Fondation thaïlandaise pour la promotion de la santé, appelé « ThaiHealth ». Cette fondation tire ses revenus d'une majoration de 2% des taxes d'accise sur le tabac et l'alcool. Ces fonds soutiennent la production de données, l'organisation de campagnes et la mobilisation sociale pour agir sur les facteurs de risque de maladie non transmissible, tels que la consommation de tabac, la consommation nocive d'alcool et le comportement sédentaire. Le revenu annuel moyen de ThaiHealth s'élève à 120 millions de dollars des États-Unis. Certaines campagnes publiques importantes financées par ThaiHealth prônent l'élimination des boissons gazeuses sucrées dans les écoles, la privation d'alcool pendant les trois mois de la retraite de la saison des pluies, et l'activité physique dans tout le pays. Le pourcentage des fumeurs de tabac est passé de 22,5% en 2001 à 18,2% en 2014. La consommation annuelle d'alcool par habitant est passée de 8,1 litres d'alcool pur en 2005 à 6,9 litres en 2014. Le pourcentage de la population adulte faisant au moins 150 minutes d'exercices aérobiques modérément intenses ou 75 minutes d'exercices aérobiques très intenses par semaine est passé de 66,3% en 2012 à 72,9% en 2017. Un mécanisme de financement spécial, une organisation transparente et responsable, et l'engagement d'organisations de la société civile et d'autres agences gouvernementales ont permis à ThaiHealth de mener ces campagnes.

## Resumen

Para facilitar la respuesta política a las enfermedades no contagiosas en Tailandia, el Parlamento aprobó en 2001 la Ley de la Fundación para la promoción de la salud. Esta ley dio lugar a la creación del organismo gubernamental autónomo, la Fundación tailandesa para la promoción de la salud, denominada ThaiHealth. La fundación recibe ingresos de un recargo del 2 % de los impuestos especiales sobre el tabaco y el alcohol. El fondo apoya la generación de pruebas, las campañas y la movilización social para hacer frente a los factores de riesgo de las enfermedades no contagiosas, como el consumo de tabaco, el consumo nocivo de alcohol y los hábitos sedentarios. De media, sus ingresos anuales ascienden a 120 millones de dólares estadounidenses. Algunas de las campañas públicas que apoya ThaiHealth van dirigidas a sacar de las escuelas las bebidas con gas azucaradas, a la abstinencia del alcohol durante la cuaresma budista de tres meses y a fomentar la actividad física en todo el país. El porcentaje de personas que consumen tabaco disminuyó del 22,5 % en 2001 al 18,2 % en 2014. El consumo anual de alcohol per cápita disminuyó de 8,1 litros de alcohol puro en 2005 a 6,9 litros en 2014. El porcentaje de población adulta que hace al menos 150 minutos de ejercicio aeróbico de intensidad moderada o 75 minutos de ejercicio aeróbico de alta intensidad por semana aumentó del 66,3 % en 2012 al 72,9 % en 2017. Un mecanismo de financiación específico, una organización transparente y responsable, así como la participación de organizaciones de la sociedad civil y otros organismos gubernamentales han permitido a ThaiHealth llevar a cabo estas campañas.

## ملخص

لتسهيل استجابة السياسات للأمراض غير المعدية في تايلند، اعتمد البرلمان قانون مؤسسة النهوض بالصحة في عام 2001. وقد أدى هذا القانون إلى إنشاء هيئة حكومية مستقلة، وهي مؤسسة النهوض بالصحة التايلندية، ويُطلق عليها اسم **ThaiHealth **. تحقق المؤسسة إيراداتها من خلال رسم إضافي بنسبة 2٪ من الضرائب المفروضة على التبغ والكحول. تدعم هذه الأموال تجميع الأدلة، والحملات، والتعبئة الاجتماعية للتعامل مع عوامل اختطار الأمراض غير المعدية، مثل تعاطي التبغ، والاستخدام الضار للكحول والسلوك المستقر. وفي المتوسط​​، تبلغ إيراداته السنوية 120 مليون دولاراً أمريكياً. بعض الحملات العامة البارزة المدعومة من مؤسسة **ThaiHealth ** موجهة للمدارس الخالية من المشروبات الغازية المحلاة؛ للامتناع عن الكحول خلال فترة الصيام البوذي التي تدوم لمدة ثلاثة أشهر؛ والنشاط البدني في أنحاء البلاد. انخفضت النسبة المئوية للأشخاص الذين يستخدمون التبغ من 22.5٪ في عام 2001 إلى 18.2٪ في عام 2014. انخفض استهلاك الفرد السنوي من الكحول من 8.1 لترات من الكحول النقي في عام 2005 إلى 6.9 لتراً في عام 2014. والنسبة المئوية للسكان البالغين الذين يمارسون ما لا يقل عن 150 دقيقة من التمارين الرياضية ذات الكثافة المعتدلة، أو 75 دقيقة من التمارين الرياضية ذات الكثافة العالية في الأسبوع، ارتفعت من 66.3٪ في عام 2012 إلى 72.9٪ في عام 2017. إن كل من آلية التمويل المخصصة، والمؤسسة التي تتمتع بالشفافية والخضوع للمساءلة، وكذلك مشاركة منظمات المجتمع المدني والوكالات الحكومية الأخرى، قد أتاحت لمؤسسة **ThaiHealth ** إدارة هذه الحملات.

## 摘要

为了促进泰国对非传染性疾病的政策响应，议会于 2001 年通过了《泰国健康促进基金会法》。该法案促成了自治政府机构，即泰国健康促进基金会（简称 ThaiHealth）的成立。该基金会的收入来自烟草和酒精消费税 2% 的附加费。该基金会支持宣传非传染性疾病风险因素（例如吸烟、酗酒与久坐不动）的证据生成、活动集会以及社会动员。平均而言，其年收入为 1.2 亿美元。ThaiHealth 举办过的著名公共活动有：倡导学校提供不含糖的碳酸饮料；在为期三个月的守夏节（佛教的四旬斋节）的戒绝酒精活动；以及全国范围内的体育活动。吸烟人群占比从 2001 年的 22.5% 降低至 2014 年的 18.2%。人均饮酒量从 2005 年的 8.1 升纯酒精降低至 2014 年的 6.9 升。每周至少进行 150 分钟中等强度或 75 分钟高强度有氧运动的成人占比从 2012 年的 66.3％ 增加至 2017 年的 72.9％。一个专门的筹资机制、一个透明负责任的组织，以及民间社会组织和其他政府机构的参与，令 ThaiHealth 得以顺利开展这些活动。

## Резюме

С целью содействия принятию мер по программе борьбы с неинфекционными заболеваниями в Таиланде парламент страны принял Закон о фонде развития здравоохранения в 2001 году. В соответствии с данным законом был создан независимый правительственный орган — Фонд развития здравоохранения Таиланда (ThaiHealth). Прибыль фонда складывается из 2%-й надбавки на табачный и алкогольный акциз. Фонд поддерживает разработку информации, проведение кампаний и мобилизацию общественности для привлечения внимания к факторам риска неинфекционных заболеваний, таким как курение табака, злоупотребление алкоголем и малоподвижный образ жизни. В среднем годовой объем поступлений составляет 120 миллионов долларов США. Среди наиболее важных общественных кампаний, поддержанных ThaiHealth, можно перечислить следующие: отказ от использования сахаросодержащих газированных напитков в школах, воздержание от алкоголя на протяжении принятого в буддизме трехмесячного поста и повышение физической активности в национальном масштабе. Доля курильщиков табака сократилась с 22,5% в 2001 году до 18,2% в 2014 году. Ежегодное потребление алкоголя на душу населения упало с 8,1 литра чистого алкоголя в 2005 году до 6,9 литра в 2014 году. Доля взрослого населения, практикующего по меньшей мере 150 минут средней интенсивности или 75 минут высокоинтенсивных аэробных упражнений в неделю, выросла с 66,3% в 2012 году до 72,9% в 2017 году. Специализированный механизм финансирования, прозрачность и подотчетность организации, а также привлечение общественных объединений и других правительственных органов позволили Фонду ThaiHealth провести эти кампании.

## Introduction

In Thailand, noncommunicable diseases were the cause of eight of the top ten disease burden in 2014.^1^ Effective policies to prevent and control these diseases are therefore needed. However, developing policies to address the risk factors for noncommunicable diseases, such as tobacco use, harmful use of alcohol and unhealthy diets, can be challenging because of the vested interests that the tobacco, alcohol and food industries have in increasing consumption.^2^ Furthermore, trade, agriculture, financial and health sectors often share the responsibility of policy development, which can lead to policy incoherence due to conflicting mandates. For example, fiscal policies, such as minimized tax and tariffs, can promote the consumption of goods and services that are in direct conflict with the government’s health goals. These fiscal policies may also contradict the World Health Organization’s (WHO’s) recommended interventions to control tobacco use, harmful use of alcohol and unhealthy diets.^3^

Challenges posed by external factors, especially globalization and trade agreements, may also hamper noncommunicable disease control efforts.^4^ For example, globalization has led to increased tobacco promotion targeting young adolescents and women living in emerging economies. Marketing aims to shape the societal norms around tobacco, by associating smoking with concepts such as “western”, “modern” and “prosperous”.^5^

In Thailand, tobacco control efforts started in 1987, with a nationwide campaign called “Run Against Tobacco”. This campaign raised sufficient public awareness on the negative health impacts of tobacco^6^ to call for legislation on tobacco control. In 1992, the Thai parliament adopted two tobacco acts, the Tobacco Product Control Act (1992) and the Non-Smoker’s Health Protection Act (1992).

The Thai Health Promotion Foundation (ThaiHealth) was established in 2001. The foundation provides its partners financial and technical support and monitors and evaluates their activities. We describe the foundation and the lessons learnt since its inception.

## Establishment of ThaiHealth

Several factors contributed to the establishment of ThaiHealth. First, the 1986 Ottawa Charter for Health Promotion recognized the challenges of sustaining adequate funding for health promotion activities.

Second, lessons learnt from tobacco control showed that key barriers to effective interventions were small annual budget allocations and the health ministry’s management of the budget. The lack of civil society organizations in programme implementation also hindered progress. 

Third, senior public health leaders in Thailand recognized the potential benefit of using dedicated tax from tobacco and alcohol as an innovative financing mechanism for health promotion. They were inspired by the experiences of the Australian health promotion foundation, VicHealth,^7^ which used tobacco tax revenue to fund health promotion interventions. The funding was made possible through the Victorian Tobacco Act. The Act endorsed a tax increase from 25% to 30% of the wholesale price of tobacco products, and the revenue from the tax increase was earmarked to VicHealth.^7^

In 1999, a group of Thai public health leaders and health professionals established a working group and called for innovative government financing to address the increasing noncommunicable disease burden. After two years of political negotiations and legislative processes, parliament enacted the Health Promotion Foundation Act in 2001 which lead to the establishment of an autonomous government body, ThaiHealth.

Thailand experienced a rapid epidemiological transition in the early 2000s, when the burden from communicable diseases, maternal and child conditions and malnutrition decreased and the noncommunicable disease burden increased.^8^

## ThaiHealth operation

**Fig. 1:**
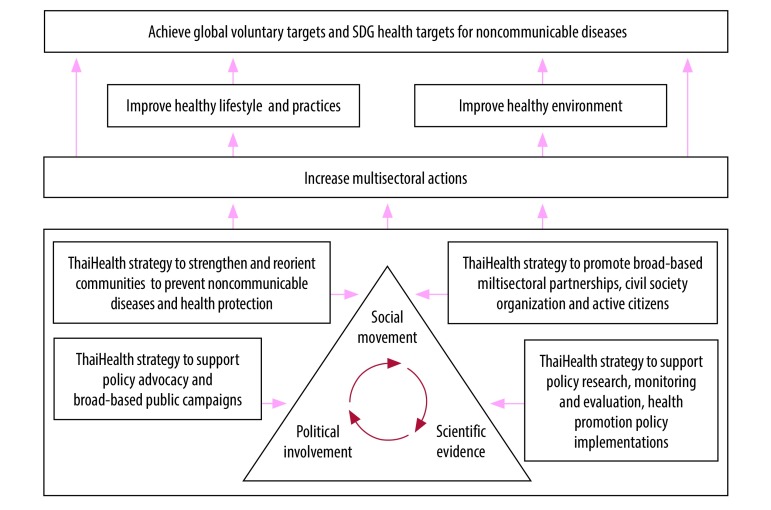
ThaiHealth contributions to targets for noncommunicable diseases and health-related sustainable development goals****

To address the multisectoral aspects of noncommunicable diseases prevention, ThaiHealth uses the “triangle that moves the mountain” approach (Fig. 1).^9^ This approach emphasizes the synergy between three factors: scientific evidence; policy decisions; and citizens and civil society organizations, who ensure accountability. This approach has previously been used for alcohol control in Thailand.^9^

ThaiHealth is set up to provide financial and technical support (including the contents of public campaigns) to its multidisciplinary network of partners who address risk factors for noncommunicable diseases: harmful use of alcohol, tobacco, unhealthy diets and sedentary behaviour. The partners range from community leaders to government agencies. ThaiHealth selects its partners through independent expert reviews of funding proposals and contracts these partners to run the programmes.

The foundation also supports monitoring and evaluation of the implementation as well as national surveys on health behaviors such as smoking, alcohol use, food consumption and physical activity.

### Governance

Two governing bodies, the governing board and the evaluation board, direct ThaiHealth. The chair of the governing board is the Prime Minister, with the Health Minister and an independent expert as the first and second vice-chair, respectively. The Cabinet appoints the governing board’s members, which consist of *ex-officio* members from nine ministries and eight independent experts from different fields. The members are appointed in their personal capacities for an initial term of three years.^10^

The evaluation board is responsible for programme and performance assessments and publishes an annual report for the governing board, which submits the report to the Cabinet, the House of Representatives and the Senate.^10^ The evaluation board consists of seven independent experts, who have been recommended by the finance minister. The Cabinet appoints experts for an initial term of three years.^10^

### Accountability

Annual external evaluations of transparency, efficiency and performance at organization, programme and project levels are published.^11^^,^^12^

### Funding and expenditure

ThaiHealth receives its funding from a 2% levy on excise taxes on tobacco and alcohol.^10^ This financing mechanism was a shift from the conventional central pooling of all sources of government revenues to the treasury, whence the treasury subsequently allocates the money to government agencies through an annual legislative process of a budget bill.

**Fig. 2:**
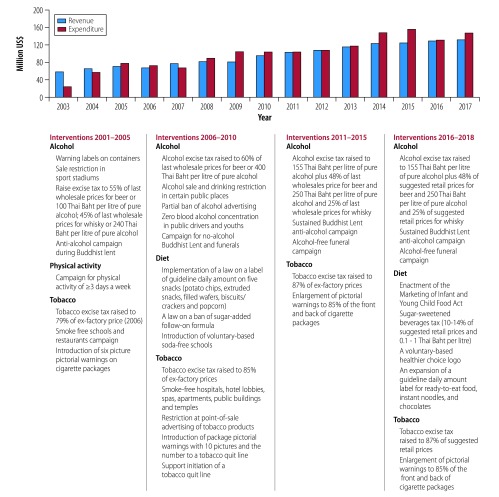
ThaiHealth revenue, expenditure and chronology of events of introducing interventions addressing the noncommunicable disease burden, 2003–2017

Since 2003, the funding has increased from 58 million United States dollars (US$) to US$ 132 million in 2017, with some fluctuations. During the same period, the expenditure has increased from US$ 23 million to US$ 147 million.^12^ Unspent funds can be retained so that surplus revenue can be used in years of higher expenditure (Fig. 2).

US$: United States dollars.

Note: 2018 conversion rate of 33 Thai Baht to US$ 1.

Between 2002 and 2017, an average 92% of the annual ThaiHealth expenditure has been allocated to health promotion programmes across the country; the remaining funds are used for operating (6%) and personnel (2%) costs.^12^

Of the US$ 129.3 million expenditure in 2017, ThaiHealth spent the largest portion (32%; US$ 41.1 million) on campaigns for tobacco, harmful use of alcohol and substance abuse, physical activity, healthy food and road safety. Healthy community strengthening programmes received 12% (US$ 15.8 million) of the funding and health literacy promotion received 9% (US$12.2 million; Table 1). These proportions have been similar in the last five years.

**Table 1:** Expenditure for the 11 programmes, ThaiHealth, 2017

Programme	Expenditure, million US$ (%)
1. Campaigns for tobacco, alcohol and substance abuse, physical activity, healthy food and road safety	41.1 (32)
2. Healthy community strengthening	15.8 (12)
3. Health literacy promotion	12.2 (9)
4. Health promotion innovation and responses to proposals^a^	10.5 (8)
5. Health promotion for vulnerable populations	10.1 (8)
6. Promotion for healthy children, young people and families	8.1 (6)
7. Public media for health advocacy	7.8 (6)
8. Health promotion in organizations and workplaces	5.8 (4)
9. Health risk control	5.6 (4)
10. Health promotion in health service system	4.5 (3)
11. Health promotion mechanism development	7.8 (6)
**Total**	**129.3 (100)**

US$: United States dollars.

^a^ Responses to innovative proposals by any organizations.

Source: ThaiHealth annual report 2016.^12^

### Campaigns and programmes

Fig. 2 presents key campaigns and programmes supported by ThaiHealth between 2002 and 2017. For more than a decade, ThaiHealth has provided support to health systems and policy research programmes, as well as policy advocacy agencies, such as the Thailand Burden of Disease studies,^13^ the Centre for Alcohol Studies and the STOPDRINK network. The Burden of Disease monitors priority risks contributing to disability adjusted life years. The Center for Alcohol Studies is responsible for generating and providing evidence to support the development of alcohol control policies^14^, such as road traffic deaths associated with alcohol consumption, estimates for the social cost of alcohol and surveillance of underage alcohol sales.^12^ The centre generated evidence for the economic case for fostering political commitment and shaping social norms towards non-tolerance of domestic and sexual violence caused by the use of alcohol.^15^ The STOPDRINK network is a non-profit civil society organization that aims to create social awareness on the harmful use of alcohol and to advocate for control policies.^16^ The centre and the network have influenced relevant government agencies to strengthen enforcement of the Alcohol Control Act 2008.^12^

The food and nutrition policy for the health promotion programme, supported by ThaiHealth, reviewed how the infant formula industry in Thailand violated the International Code of Marketing of Breast-milk Substitutes.^17^ Working with civil society organizations, the programme advocated for national legislation related to the Code. This advocacy led to the Control of Marketing of Infant and Young Child Food Act in 2017. The programme was opposed by certain paediatricians and the medical council, who argued the downsides of breastfeeding by quoting a study from Nepal^18^ claiming that “prolonged breastfeeding beyond 12 months results in stunting.” The programme officers responded to this argument with the evidence that multiple factors contribute to stunting, such as low socioeconomic status, low maternal education, poverty, inadequate and inappropriate supplementary feeding practices. This programme also worked with the network for schools free from sweetened carbonated beverages to generate evidence for the introduction of a sugar-sweetened beverage tax,^19^ despite strong opposition from the beverage industries (Box 1).

Box 1 **Opposition from beverage industries on introduction of sugar-sweetened beverage tax in Thailand**

To address the increasing trends in the prevalence of overweight and obesity, the National Legislative Council introduced a sugar-sweetened beverage tax in September 2017.^20^ Before the legislative process, the Thai Beverage Industry Association and its alliance lobbied intensively to stop the introduction of such tax by sending an open letter to the chair of National Reform Steering Assembly. This assembly is a decision-making body for public policy formulation before submission to the National Legislative Council. The association’s arguments against sugar-sweetened beverage tax were:

- the tax is not effective since there are many unhealthy substitutes to sugary drinks;
- the tax is unfair to the ready-to-drink beverage sector since other products, in particular, non-ready-to-drink products, are not covered;
- other unhealthy foods such as confectionary are not regulated;
- the tax will have a negative impact on the poor who consume these products;
- the tax will have negative impacts on sugarcane farmers and other allied industries such plastic bottle production and logistics; and
- the tax violates consumers’ rights.

Moreover, after the National Reform Steering Assembly adopted the tax proposal; they officially asked to participate, but were not allowed due to conflict of interest, in the development of the sugar-sweetened beverage tax implementation plan.

During the development of the tax proposal, a leading Thai newspaper published several articles that challenged the reasoning that sugar-sweetened beverage tax can reduce consumption and have positive impact on childhood obesity. Articles also argued that industry may absorb the tax by not increasing retail prices to maintain market share and consumption.

A few notable ThaiHealth-supported public campaigns include: schools free of sweetened carbonated beverages; alcohol abstinence during three-month Buddhist lent; and physical activity. The latter have been nationwide campaigns for both marathons and fun runs, in which people participate for their own enjoyment rather than to compete. In many provinces across Thailand, campaigns have promoted an active lifestyle and expansion of bike lanes.^11^

Alcohol-free Buddhist lent is an annual evidence-based campaign that uses Buddhism’s five moral precepts; to refrain from harming living things, stealing, sexual misconduct, lying and intoxication. The campaign is thus culturally and religiously acceptable. The campaign encourages three months abstention from alcohol. Interestingly, there is no resistance from the alcohol industry.^21^

Using evidence from social marketing techniques, foundation-supported programmes have successfully shaped social norms of no-alcohol gifts during celebrations such as birthdays and New Year. The campaign uses the slogan “Giving alcohol (as a gift) is a curse (to the recipient).” Alcohol gift-giving reduced from 30.5% of total gift-giving in 2008 to 13.1% in 2012.^12^ However, campaigns to promote alcohol-free funerals and Thai New Year festivals and “Drink, don’t drive” advertising to prevent traffic deaths have been less successful.^22^

### Other collaborations

**Fig. 3:**
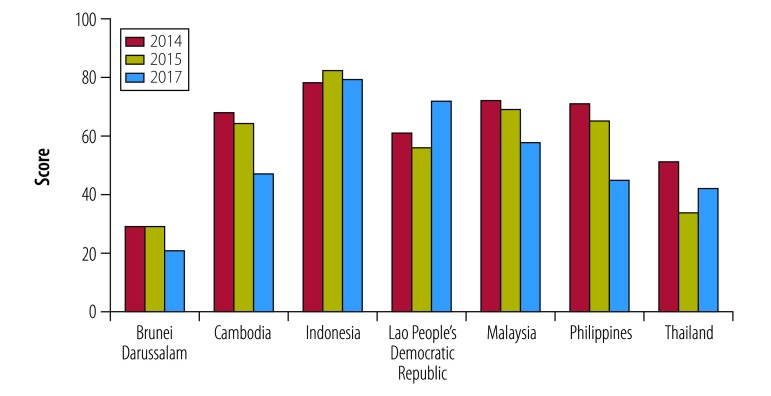
Trends in tobacco industry interference index in seven countries, 2014, 2015, 2017

Evidence exists that the tobacco industry interferes with the development of national control policies and with the implementation of WHO tobacco control policies and programmes.^23^^–^^25^ ThaiHealth works closely with the Southeast Asia Tobacco Control Alliance, which publishes an annual tobacco industry interference index. The index consists of 20 indicators divided into seven categories: (i) level of participation in policy development; (ii) so-called corporate social responsibility activities; (iii) benefits to the tobacco industry; (iv) forms of unnecessary interaction; (v) transparency; (vi) conflict of interest; and (vii) preventive measures.^25^ In 2017, Brunei Darussalam and Thailand showed the lowest levels of tobacco industry interference of the 14 countries studied (Fig. 3).^25^

Data source: Southeast Asia Tobacco Control Alliance.^25^^,^^26^

The International Trade and Health Programme, supported by ThaiHealth, has generated evidence to inform policy decisions on trade agreements that could have negative health consequences. The evidence was used during the negotiation of a free trade agreement between Thailand and the European Union.^27^

## Outcomes

Fig. 2 presents political decisions based on evidence generated by ThaiHealth partners. Despite opposition from industries, ThaiHealth and its networks have helped to implement many of WHO’s recommended interventions and other measures to address noncommunicable diseases.^12^

### Policy outcomes

#### Alcohol

Between 2001 and 2005, the government adopted warning labels on alcohol products and restricted the sale of alcohol in sport stadiums.^28^ From 2006 to 2010, the government restricted alcohol sales and drinking in additional settings; introduced a partial ban of alcohol advertising; and blood alcohol limits of zero for drivers of public transport and those under 20 years of age. The alcohol excise tax has been regularly revised and raised since 2001.

#### Tobacco

ThaiHealth and the Action on Smoking and Health Foundation have been advocating for tighter tobacco control measures, such as increasing the area of pictorial warning on cigarette packs; increasing taxes; extending non-smoking areas; and banning electronic cigarettes.

Between 2001 and 2005, the government introduced policies to ban smoking in schools and restaurants and enforced six choices of pictorial warnings.^29^ Between 2006 and 2010, tobacco promotion was restricted at point-of-sale, pictorial warnings with 10 pictures and a tobacco quit line were introduced. The smoking ban was expanded to hospitals, hotel lobbies, spas, public buildings and temples. In 2014, pictorial warnings were expanded to 85% of front and back package area. In 2016, the parliament adopted a major tobacco tax reform, including a specific tax rate and an added-value tax. Taxes are now calculated based on suggested retail prices.

#### Diet and physical activity

The efforts to promote healthy diets and physical activity have been less than for tobacco and alcohol control. From 2006 to 2010, two interventions aimed to address over-consumption of sugar; a ban on sugar-added formula and a voluntary sugar-sweetened-beverage-free school programme.^30^ In 2011, a mandatory label of a daily amount guideline on five snacks was adopted. From 2016, the government introduced sugar-sweetened beverage taxation based on sugar content in beverages; the tax rate increases with higher sugar content,^31^ and voluntary implementation of the logo “healthier choice” on food items with low fat, low sugar and low salt.^32^ The daily amount guideline label was expanded to appear on five food product categories: snacks; chocolates; chilled or frozen ready-to-eat meals; bakery products; and semi-processed foods.^33^

### Health outcomes

**Fig. 4:**
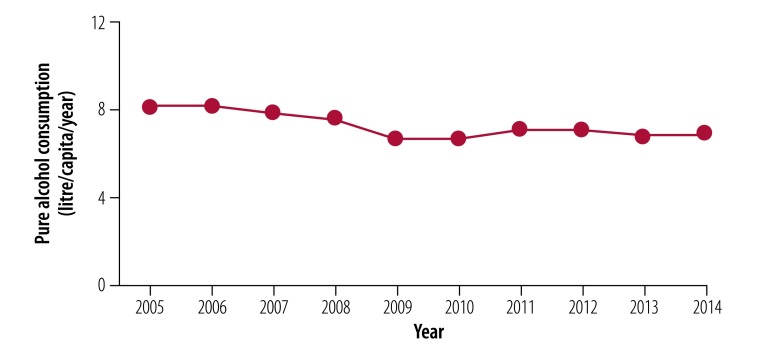
Trend of annual per capita alcohol consumption, Thailand, 2005–2014

The health outcomes described here are due to a collective effort to which ThaiHealth has contributed over the past 15 years. The percentage of people using tobacco decreased from 22.5% in 2001 to 18.2% in 2014.^34^ Total annual per capita alcohol consumption, after fluctuations between 2001–2005, decreased from 8.1 litres of pure alcohol in 2005 to 6.9 litres in 2014 (Fig. 4).^35^

Addressing overweight and obesity have been less successful, despite the fact that the percentage of adults achieving sufficient physical activity (defined as at least 150 minutes of moderate-intensity aerobic exercise per week or 75 minutes of vigorous-intensity aerobic exercise per in a week) increased from 66.3% of total adults in 2012 to 72.9% in 2017.^36^ Between 1992 and 2014, results from nationally-representative surveys showed that the prevalence of overweight among Thai adults increased from 18.2% to 37.5%.^37^^,^^38^ The prevalence increased 32.4% between 1992 and 1997 and 2.7% between 2009 and 2014.^37^^–^^40^ The increase in overweight could be partly explained by an increase in the consumption of soft drinks, which grew by 23.8% between 2010 and 2015, from 93.3 to 115.6 litres per capita, respectively.^41^ Also, the increased market penetration of food high in fat, salt and sugar, which influence food choices among Thai people,^42^ could be a potential factor in the increase in the prevalence of overweight. 

## Lessons for policies and practices

ThaiHealth’s role as an innovative funding agency has been a key factor in providing strategic support for health promotion, as the foundation can contract civil society organizations for programme implementation or campaigns. A few lessons emerge for those who are interested in setting up a similar funding approach, in addition to the lessons presented in a previous publication.^43^

### Financing source and use

The ThaiHealth experience demonstrates that dedicated funding combined with flexibility in supporting civil society organizations and the use of performance-based contracts are essential to success. In contrast, the health ministry’s budget is usually implemented through its affiliated health facilities. One of the advantages of involving civil society organizations is in their capacities to challenge tobacco, alcohol and unhealthy food industries.

The board members comprise representatives from relevant sectors, which enables ThaiHealth to work with diverse partners across institutions and to reduce bureaucratic rigidity. To ensure transparency, members of the board, committees, working groups and the secretariat must declare any conflict of interests. The annual performance reports ensure transparency and accountability of the use of public resources.

### Establishment

ThaiHealth faced difficulties when several Thai economists, not in favour of establishing a dedicated fund, argued that the fund would undermine financial discipline as the government and parliament would lose control over revenue and expenditure. They also argued that the establishment of such a fund sets a precedent for other sectors to demand the same. To date, there is no legislation for another dedicated fund. With strong leadership from the Prime Minister and the Finance Minister, these difficulties were addressed, showing the importance of the highest level of political commitment when establishing a dedicated fund for health promotion.

### Amendment of the Act

ThaiHealth may encounter further difficulties because of a proposed amendment to the Health Promotion Foundation Act in 2018.^44^ The amendment focuses on four areas: (i) the maximum ceiling of the fund shall not exceed US$ 121 million and any additional revenue from tax will be centralized to the treasury; (ii) the criteria for using the fund shall be approved by the finance ministry, adding an additional approval step; (iii) reduction in the number of *ex-officio* members of the governing board from nine to seven ministries, and independent experts from eight to six; and (iv) the amendment of the three-year term of independent experts from unspecified numbers of renewals to renewal only once. In 2017 the size of the fund was US$ 132 million and hence a ceiling would constrain the operation of ThaiHealth in the long term

The civil society organizations describe the amendment of the Act in public media as a struggle between the civic movement for health protection of the people and conservative bureaucrats. The civil society organizations argue that the requirement of an approval of the funding by the finance ministry will result in excessive and complex official procedures, which most likely will lead to delay or even inaction and potential political interference.^45^^–^^47^ In October 2018, the amendment of the Act initially went through four regional public hearings. As of February 2019, the national public hearing is pending to summarize first findings. The consolidated findings will be submitted to the Cabinet for endorsement, then the Office of the Council of State will comment on the amendment before the National Legislative Assembly starts the legislative processes.
